# Using Video Feedback Through Smartphone Instant Messaging in Fundamental Nursing Skills Teaching: Observational Study

**DOI:** 10.2196/15386

**Published:** 2019-09-05

**Authors:** Xiaoxian Yang, Ri-Hua Xie, Si Chen, Wei Yu, Yan Liao, Daniel Krewski, Shi Wu Wen

**Affiliations:** 1 School of Nursing Wuxi Taihu University Wuxi China; 2 Department of Nursing Nanhai Hospital Southern Medical University Foshan China; 3 General Practice Center Nanhai Hospital Southern Medical University Foshan China; 4 Ottawa Hospital Research Institute Ottawa, ON Canada; 5 McLaughlin Centre for Population Health Risk Assessment Faculty of Medicine University of Ottawa Ottawa, ON Canada; 6 School of Epidemiology and Public Health Faculty of Medicine University of Ottawa Ottawa, ON Canada; 7 Ottawa Hospital Research Institute University of Ottawa Ottawa, ON Canada; 8 Department of Obstetrics & Gynecology Faculty of Medicine University of Ottawa Ottawa, ON Canada

**Keywords:** video feedback, smartphone, mobile phone, student nurses, fundamental nursing skill, teaching

## Abstract

**Background:**

Video feedback has been shown to be an effective teaching tool that can improve student learning when having them view their own performance. However, the literature on the effect of integrating smartphones with video feedback in fundamental nursing skills teaching is sparse.

**Objective:**

This study aimed to explore the potential effects of video feedback through smartphone-based instant messaging on teaching undergraduate nursing students fundamental nursing skills.

**Methods:**

We conducted a study on teaching fundamental nursing skills to 6 classes of second-year undergraduate nursing students. In 2 classes (the intervention group), the instructor elected to use smartphone-based video feedback to facilitate teaching; instructors in the other 4 classes (the control group) elected to use routine methods of teaching without video feedback. Scores from the final examination, in-class assignments, and the General Self-Efficacy Scale questionnaire were collected and compared between the two groups. Multiple linear regression analysis was performed to estimate the independent effect of video feedback after adjusting for gender, age, and prior experience in the use of WeChat/QQ in learning applications. An ad hoc questionnaire was used for student evaluation of the novel smartphone-based video feedback teaching method.

**Results:**

A total of 195 nursing students (65 in the video feedback group and 130 in the control group) completed the study and were included in the final analysis. Mean and standard deviation of scores on the final examination, bed making, aseptic procedure, vital signs measurement, and oxygen therapy were 91.29 (SD 2.36), 90.52 (SD 3.18), 93.23 (SD 3.16), 91.65 (SD 4.21), and 92.06 (SD 3.58), respectively, in the video feedback group and 89.99 (SD 3.12), 81.71 (SD 8.63), 87.12 (SD 5.50), 87.45 (SD 8.00), and 90.37 (SD 6.36), respectively, in the control group (differences were statistically significant). The mean and standard deviation of scores for assignments in catheterization and enema and General Self-Efficacy Scale were 89.69 (SD 3.22), 91.14 (SD 3.15), and 24.52 (SD 5.35), respectively, in the video feedback group and 88.82 (SD 7.48), 90.79 (SD 6.08), and 24.50 (SD 6.16), respectively, in the control group (differences were not statistically significant). The majority (over 98%) of nursing students were satisfied with this smartphone-based video feedback teaching method.

**Conclusions:**

Video feedback through smartphone-based instant messaging may be an effective way to improve nursing students’ academic performance and professional skills.

## Introduction

Video feedback has been demonstrated to be an effective teaching tool that can improve student skills by having them view their own performance on video [1]. Students can identify what they did well, what they did not do so well, and what they could improve through video feedback in nursing skills training [2]. A meta-analysis showed that video feedback has a positive effect on learning [1]. However, video feedback with standard equipment for large classroom teaching is not convenient and lacks flexibility. It is therefore important to find a way to improve the process for video feedback in medical education.

Smartphones are very popular with the youth and could be exploited to improve learning as a consequence of personal behavior changes [3,4]. In recent years, China has developed popular apps such as WeChat and QQ, which support instant text messaging and voice and video calling via smartphones. WeChat and QQ are the most popular personal communication tools used by university and college students in China, making them attractive options for implementing enhanced teaching methods in medicine and nursing [5,6]. The penetration rate of both smartphone and WeChat/QQ use is almost 100% among university and college students, providing a convenient basis for integrating the video feedback in nursing skills training [7,8].

Attempts have been made to use smartphones to achieve positive results for a range of medical and nursing education issues, including coordination supporting among groups [7], theory and practice integrating [9], student participation/engagement [10], and communication skill enhancement [7]. Nursing skills practice could be recorded in video format by students with their smartphones and then sent to instructors via instant messaging. The instructors, in turn, could provide comments and suggestions on student performance, providing rapid content-related feedback. As the literature on the potential benefits of video feedback on the teaching of fundamental nursing skills is sparse, our study was conducted to evaluate the effects of video feedback through smartphone-based instant messaging on teaching fundamental nursing skills.

## Methods

### Study Participants

This study was carried out in Wuxi Taihu University School of Nursing between October 1 and November 30, 2018. There were 6 classes of second-year undergraduate nursing students who were taking the fundamental nursing skills course during that semester. There were 3 instructors, each of whom taught 2 classes with no overlap/exchanges/substitutions throughout the entire semester. The instructors were qualified nursing educators with the same seniority. At the beginning of the semester, an instructor for 2 of the 6 classes elected to use smartphone-based video feedback to facilitate her teaching. One author of the research team approached the instructors with a request to undertake a study to evaluate the effect of this new method of teaching. After the instructors agreed to participate in the study, an approval from the institutional review board of Taihu University was obtained. Students from the 2 classes whose instructor planned to use the smartphone-based video feedback comprised the intervention group (video feedback group), while students from other 4 classes whose instructors planned to use routine teaching methods (no video feedback) formed the control group. All students provided written informed consent to participate in the study. Fundamental nursing skills taught in this study were performed on manikin simulators and included bed making, aseptic procedures, vital signs measurement, oxygen therapy, catheterization, and enema administration. Students from both groups were asked to practice and complete one assignment for each skill after class. In the video feedback group, the instructor first explained the basic material and demonstrated the basic procedures in the classroom. Afterward, the nursing students worked in groups of 3 or 4 to record videos of each other on their smartphones. Each video lasted 5 to 15 minutes and took more than 10 minutes to upload and download. The students then sent the videos to the instructor via instant messaging for evaluation and feedback on their nursing practices (the instructor spent 5 to 15 minutes going through each video).

### Outcome Measures

Scores on the final examination and on the 6 nursing skills assignments (bed making, aseptic procedure, vital signs measurement, oxygen therapy, catheterization, and enema) were the main outcomes of interest. Teaching and evaluation at Wuxi Taihu University School of Nursing were performed by different faculty members (faculty members involved in teaching cannot evaluate their own students).

The General Self-Efficacy (GSE) Scale [11], which is designed to assess optimistic self-beliefs related to coping with a variety of demands in life, was a secondary outcome. The GSE Scale comprises 10 questions, each of which is scored on a 4-point Likert scale (1=not at all true, 2=hardly true, 3=moderately true, 4=exactly true). Total score ranges between 10 and 40. This scale was originally developed by Schwarzer and Jerusalem [11] and has been used and evaluated in several populations and cultures. The Chinese version of the GSE Scale has been used in college and university students in China [12]. Its Cronbach alpha coefficient is 0.87, the retest reliability is 0.83, the half-fold reliability is 0.90, and the validity ranges from 0.60 to 0.77 [13].

Finally, an ad hoc questionnaire was developed to measure the students’ own evaluations of the video feedback teaching method in the video feedback group. The questionnaire has 6 items with 5 response categories expressing the degree of agreement with each item.

### Data Analysis

We first compared the distribution of baseline characteristics between the intervention and control groups. We then compared the scores on the final examination, the 6 nursing skills assignments, and GSE scores between the two groups. Multiple linear regression analysis was conducted to estimate the independent effect of video feedback on student performance after adjusting for gender, age, and prior experience in the use of WeChat/QQ in learning applications. Two-sided tests were used in all comparisons between the two study groups. Finally, student evaluations of the video feedback teaching method were presented. All analyses were performed using SAS v9.4 (SAS Institute Inc).

## Results

### Baseline Characteristics of Study Participants

A total of 195 nursing students (65 in the video feedback group and 130 in the control group) completed the study and were included in the final analysis. Table 1 shows the distribution of baseline characteristics of the two groups. There were no differences in gender, age, or prior experience in the use of WeChat/QQ in learning applications between the two groups.

### Comparison of Scores in the Six Nursing Skills Between the Intervention and Control Groups

Table 2 presents the mean and SD of the final examination scores and aggregate and individual scores for the 6 indicators of nursing skills. Scores on the final examination, bed making, aseptic procedure, vital signs measurement, and oxygen therapy were significantly higher in the video feedback group than in the control group. No significant differences in scores on catheterization and enema between the two study groups were observed.

### Comparison of Scores on the General Self-Efficacy Scale Between the Intervention and Control Groups

Table 3 compares the mean and standard deviation of the total GSE score and the 10 individual items comprising the GSE Scale between the two study groups. A total of 148 students (48 students in video feedback group and 100 students in control group) provided answers to the questionnaire. Mean and SD of the total scores for GSE Scale were 24.52 (SD 5.35) in the video feedback group and 24.50 (SD 6.16) in control group (differences in the total score and individual item scores were not statistically significant).

**Table 1:** Comparison of baseline characteristics of intervention and control groups.

Characteristics		Video feedback group (n=65)	Control group (n=130)	*P* value
**Gender, n (%)**				.33
	Male	5 (7.7)	16 (12.3)	
	Female	60 (92.3)	114 (87.7)	
Age (years), mean (SD)		19.65 (0.82)	19.54 (0.75)	.34
**Prior experience in the use of WeChat/QQ in learning applications, n (%)**				.18
	Yes	22 (33.9)	57 (43.9)	
	No	43 (66.1)	73 (56.1)	

**Table 2:** Comparison of scores in the 6 nursing skills between the intervention and control group.

Test scores	Video feedback group (n=65), mean (SD)	Control group (n=130), mean (SD)	Crude mean difference (95% CI)	Adjusted mean difference^a^ (95% CI)
Skill 1: bedmaking	90.52 (3.18)	81.71 (8.63)	8.82 (6.63, 11.00)	8.82 (6.67, 10.97)
Skill 2: aseptic procedure	93.23 (3.16)	87.12 (5.50)	6.12 (4.66, 7.57)	6.12 (4.66, 7.57)
Skill 3: vital signs measurement	91.65 (4.21)	87.45 (8.00)	4.19 (2.10, 6.28)	4.19 (2.12, 6.27)
Skill 4: oxygen therapy	92.06 (3.58)	90.37 (6.36)	1.69 (0.02, 3.37)	1.69 (0.01, 3.38)
Skill 5: catherization	89.69 (3.22)	88.82 (7.48)	0.87 (–1.04, 2.78)	0.87 (–1.02, 2.76)
Skill 6: enema	91.14 (3.15)	90.79 (6.08)	0.35 (–1.24, 1.93)	0.35 (–1.21, 1.90)
Average: 6 skills	91.38 (2.10)	87.71 (4.43)	3.67 (2.53, 4.82)	3.67 (2.54, 4.81)
Average: final examination	91.29 (2.36)	89.99 (3.12)	1.30 (0.43, 2.17)	1.30 (0.44, 2.16)

^a^Adjusted for gender, age, and prior experience in the use of WeChat/QQ in learning applications.

**Table 3:** Comparison of scores of the General Self-Efficacy (GSE) Scale between the intervention and control groups.

GSE Scale scores	Video feedback group (n=48), mean (SD)	Control group (n=100), mean (SD)	Crude mean difference (95% CI)	Adjusted mean difference^a^ (95% CI)
Item 1: I can always manage to solve difficult problems if I try hard enough.	2.78 (0.72)	2.82 (0.74)	–0.05 (–0.30, 0.21)	–0.05 (–0.31, 0.21)
Item 2: If someone opposes me, I can find the means and ways to get what I want.	2.50 (0.80)	2.47 (0.67)	0.03 (–0.22, 0.28)	0.03 (–0.22, 0.28)
Item 3: It is easy for me to stick to my aims and accomplish my goals.	2.00 (0.80)	2.15 (0.74)	–0.15 (–0.41, 0.11)	–0.15 (–0.41, 0.11)
Item 4: I am confident that I could deal efficiently with unexpected events.	2.21 (0.74)	2.29 (0.71)	–0.08 (–0.33, 0.17)	–0.08 (–0.34, 0.17)
Item 5: Thanks to my resourcefulness, I know how to handle unforeseen situations.	2.23 (0.78)	2.23 (0.71)	–0.00 (–0.25, 0.25)	–0.00 (–0.26, 0.25)
Item 6: I can solve most problems if I invest the necessary effort.	2.79 (0.71)	2.71 (0.69)	0.08 (–0.32, 0.16)	0.08(–0.32, 0.16)
Item 7: I can remain calm when facing difficulties because I can rely on my coping abilities.	2.63 (0.76)	2.65 (0.70)	–0.02 (–0.28, 0.23)	–0.02 (–0.27, 0.22)
Item 8: When I am confronted with a problem, I can usually find several solutions.	2.44 (0.74)	2.43 (0.69)	0.01 (–0.24, 0.25)	0.01 (–0.24, 0.25)
Item 9: If I am in trouble, I can usually think of a solution.	2.69 (0.72)	2.55 (0.63)	0.14 (–0.09, 0.37)	0.14 (–0.09, 0.36)
Item 10: I can usually handle whatever comes my way.	2.25 (0.79)	2.22 (0.79)	0.03 (–0.24, 0.30)	0.03 (–0.24, 0.30)
Total score	24.52 (5.35)	24.50 (6.16)	0.02 (–1.97, 1.93)	0.02 (–1.98, 1.94)

^a^Adjusted for gender, age, and prior experience in using WeChat/QQ in learning applications.

### Student Evaluations of Video Feedback

Table 4 presents student evaluations of the smartphone-based video feedback teaching method. Of the students who provided answers to the questionnaire, 98% (54/55) of nursing students were satisfied with the smartphone-based video feedback. Many of the nursing students strongly agreed that the video feedback teaching method can improve skill proficiency (32/55, 58%), practice passion (28/55, 51%), learning interest (27/55, 49%), learning effectiveness (31/55, 56%) and autonomous learning ability (32/55, 58%).

**Table 4:** Student evaluations of video feedback in the video group (n=55).

Item	Strongly agree, n (%)	Agree, n (%)	Uncertain, n (%)	Disagree, n (%)	Strongly disagree, n (%)
Improved skill proficiency	32 (58)	21 (38)	2 (4)	0 (0)	0 (0)
Improved practice passion	28 (51)	25 (45)	2 (4)	0 (0)	0 (0)
Improved learning interest	27 (49)	24 (44)	4 (7)	0 (0)	0 (0)
Improved learning effectiveness	31 (56)	24 (44)	0 (0)	0 (0)	0 (0)
Improved autonomous learning ability	32 (58)	20 (36)	3 (5)	0 (0)	0 (0)
Satisfied with the smartphone-based video feedback method	35 (63)	19 (34)	1 (1)	0 (0)	0 (0)

## Discussion

### Principal Findings

Our study found that video feedback through smartphone-based instant messaging may have the potential to improve the performance of nursing students in fundamental nursing skills, especially with respect to skills related to bed making, aseptic procedure, vital signs measurement, and oxygen therapy. Overall, most nursing students were satisfied with the smartphone-based video feedback teaching method. Although the study failed to demonstrate an improvement in overall self-efficacy, nursing students perceived that their interests and autonomous learning abilities had been improved.

### Strengths

To our knowledge, this is the first study incorporating smartphone instant messaging with video feedback in fundamental nursing skills teaching. Although the effect of video feedback in medical education has been well established, the need for standard equipment in regular video feedback makes this teaching method not convenient to some extent. Through smartphone-based video messaging, students were able to record the videos at a time and place that was convenient to them and send their videos to the instructor for timely and precise feedback. In Wuxi Taihu University School of Nursing, faculty members not involved in the teaching of the particular course acted as the evaluators of student performances, thereby avoiding bias in the assessment. In contrast to Western countries, Chinese universities admit students as high school graduates according to their performance on the National University/College Entrance Examination. Top-ranked universities have priority to admit students with higher scores on the examination and superior academic performance in high school. Leading national universities recruit top students from all provinces across the country, while local universities like Wuxi Taihu University mainly recruit students locally. Once admitted, students are assigned to different classes by the university administration in a somewhat random fashion. In our study, students from the intervention and control groups were similar in gender, age, and previous life experience, so any differences between the groups should be attributable to the intervention and not to inherent differences between the two groups. The most important previous life experience relevant to this study is the prior experience using WeChat/QQ messaging in learning, which was not different between the two groups (Table 1). We used multiple regression analysis in the comparison of outcomes between the 2 study groups to adjust for age, gender, and previous life experience, ensuring no residual confounding in the comparison. The skills were performed on mannikins, so there were no ethical concerns related to videography.

### Limitations

We acknowledge limitations of this study. First, whether to use the smartphone-based video feedback was a choice by the course instructor. Although all 3 instructors are qualified nursing educators with the same seniority, the instructor who elected to use smartphone-based video feedback to facilitate her teaching may be more motivated and this may have resulted in better quality in her teaching. Because this was not a randomized controlled trial, we cannot be sure about this source of bias. Second, the answers to some of the GSE Scale questions may be somewhat inaccurate and imprecise because some of the participants completed the questionnaire several weeks after the course was over. Thus, recall bias may exist. Third, the questionnaire for student evaluation of the smartphone-based video feedback was developed on an ad hoc basis without formal validation or reliability assessment. Fourth, although faculty members who evaluated student performance did not participate in teaching the course, they were from the same school and knew who the course instructor was. As a result, the skills evaluation could not be considered entirely blind. Fifth, there may be a chance that some students in the intervention group may not have actually done the video or the instructor may not have actually sent feedback to some students. Either way, actual effect of video feedback may have been diluted. Unfortunately, we did not collect these data and could not assess the impact of the quality of video feedback on the observed effect. Sixth, the time needed for video uploading and downloading depended on the network speed and could have been frustrating for students and instructors alike if the network was slow.

### Implications

This study explored a method of improving fundamental nursing skills teaching for undergraduate nursing students. It integrated smartphone technology, a mobile app, and video feedback in facilitating teaching. Smartphone-based feedback could offer a novel, flexible study method, and the feedback could allow participants to know whether they are performing well or not [14]. Students could identify problems or errors in their performance while reviewing their video and then repeat the procedures in the correct manner. This learning experience is conducive to deepening student understanding of clinical skills practice, empowering students to standardize their own skills practice and explore the limits of their own skills and abilities. Problems or errors encountered in video practice could be sent to the instructor promptly for rapid feedback and correction. In the future, application of these skills could be further improved and the tasks and procedures standardized to optimize operational performance in clinical work. This method could also increase the frequency of practice after class, which is a key point of improving nursing skills. Thus, smartphone-based instant messaging video feedback could improve fundamental nursing skills for nursing students, consistent with a previous study in Korea [15]. Of the 6 skills assessed, smartphone-based video feedback had a stronger effect on skills in bed making, aseptic procedure, vital signs measurement, and oxygen therapy. The reasons for the lack of significant improvement in catheterization and enema administration by video feedback are unclear. Catheterization and enema administration were more complicated than the other procedures, and they were the last two skills to be learned in the semester. We speculate that students may have limited time to prepare video and practice in the end of semester.

Self-efficacy is related to one’s beliefs as to whether or not they are capable of completing a certain task [16]. Studies have demonstrated that general self-efficacy is positively correlated with self-learning ability [17], indicating that improving self-efficacy could encourage nursing students to learn by themselves [18]. Using a smartphone [19] or a personal digital assistant [20] in nursing education could improve self-efficacy because both have the ability to meet their unique needs and improve confidence while learning. However, our study failed to find an improvement in self-efficacy. Some of the participants completed the GSE Scale questionnaire several weeks after the course was over; therefore, they may have not answered the questions accurately and precisely. It is also possible that one curriculum may not be sufficient to improve self-efficacy. As shown in Table 4, the majority of students perceived that the smartphone-based video feedback teaching method could improve their interest, effectiveness, and capacity of autonomous learning, which may lead to improvement in their performance on the final examination and assignments of skills, despite the lack of a significant improvement in self-efficacy.

Most students were satisfied with the smartphone-based video feedback teaching method, a finding consistent with a previous study [21]. A systematic review of the use of mobile technology in undergraduate education also showed that nursing faculty members have become more interested in incorporating such technologies into their teaching strategies [7]. In our study, the majority of students strongly agreed that video feedback could improve the proficiency of nursing skills and autonomous learning. The nursing students may become more motivated to learn when these technologies are incorporated in education [14]. The attitudes of nursing students toward an instant message–based video feedback teaching paradigm shows that this teaching method is both feasible and acceptable. It would be informative to undertake a formal qualitative evaluation of both instructors and students to further explore attitudes toward and acceptance of this new method of teaching. It should be pointed out that although it is easy for instructors to go through the videos and video feedback may improve quality of teaching, its use may increase instructor workload with respect to reviewing videos and sending feedback. On the other hand, it may reduce the workload associated with other aspects of teaching by reducing the need for face-to-face consultations. These issues need to be further explored and considered by teachers and school administrators before smartphone-based video feedback is widely implemented in nursing teaching curricula.

### Conclusions

Our study suggests that the use of video feedback through smartphone-based instant messaging may be an effective way to improve nursing students’ overall performance and skills. This novel teaching modality makes use of relatively inexpensive smartphone technology, which is now almost universally available and familiar to the millennials who will become tomorrow’s health professionals. Extending the use of smartphone-based video feedback teaching techniques more broadly across multi-year academic curricula and other areas of health sciences could lead to even better results than those observed in this limited study, including not only increased performance but increased self-efficacy.

## Abbreviations

GSE Scale: General Self-Efficacy Scale
